# Spatial organization of *Gardnerella* species, *Prevotella bivia*, and *Fannyhessea vaginae* in the bacterial vaginosis biofilm

**DOI:** 10.1128/iai.00630-25

**Published:** 2026-01-22

**Authors:** Sheridan D. George, Megan H. Amerson-Brown, Lúcia G. V. Sousa, Alexa H. Rinehart, Ashutosh Tamhane, Ashleigh N. Riegler, Sixto M. Leal, John W. Lammons, Jacob H. Elnaggar, Keonte J. Graves, Paweł Łaniewski, Melissa M. Herbst-Kralovetz, Christopher M. Taylor, Nuno Cerca, Christina A. Muzny

**Affiliations:** 1 Division of Infectious Diseases, Department of Medicine, University of Alabama at Birmingham 164494, Birmingham, Alabama, USA; 2 Division of Laboratory Medicine, Department of Pathology, University of Alabama at Birmingham 189178, Birmingham, Alabama, USA; 3 Centre of Biological Engineering (CEB), Laboratory of Research in Biofilms Rosário Oliveira (LIBRO), University of Minho 56059, Braga, Portugal; 4 Division of Nephrology, Department of Medicine, University of Alabama at Birmingham 164494, Birmingham, Alabama, USA; 5 Department of Microbiology, Immunology, and Parasitology, Louisiana State University Health Sciences Center 12258, New Orleans, Louisiana, USA; 6 Department of Basic Medical Sciences, College of Medicine – Phoenix, University of Arizona 204788, Phoenix, Arizona, USA; University of California San Diego School of Medicine, La Jolla, California, USA

**Keywords:** *Gardnerella *species, *Prevotella bivia*, *Fannyhessea vaginae*, BV biofilm, vaginal microbiome, biofilm, bacterial vaginosis

## Abstract

Key bacterial vaginosis (BV)-associated bacteria implicated in biofilm formation include *Gardnerella* species, *Prevotella bivia*, and *Fannyhessea vaginae*. We investigated their spatial organization in the BV biofilm over time from longitudinal vaginal specimens obtained from women with incident BV (iBV) using peptide nucleic acid-fluorescence *in situ* hybridization (PNA-FISH). Heterosexual women with optimal vaginal microbiota self-collected vaginal specimens twice daily for 60 days or until iBV development (Nugent score 7–10 on ≥4 consecutive specimens). Women who developed iBV were matched to healthy controls by age, race, and contraceptive method. *Gardnerella* spp., *P. bivia*, and *F. vaginae* were quantified using PNA-FISH 2 days pre-iBV, the day of iBV, and 2 days post-iBV across five optical layers (*z*, *z* + 2, z + 4, *z* + 6, and *z* + 8 μm). Total counts of all three bacterial species were significantly higher on the day of iBV compared to 2 days pre-iBV (*P* = 0.011) and remained elevated 2 days post-iBV. Across most layers and time points, pooled mean *Gardnerella* spp. counts were significantly higher than *F. vaginae* counts (*P* ≤ 0.022-0.0003). On the day of iBV and 2 days post-iBV, pooled mean counts of *Gardnerella* spp. and *F. vaginae* progressively increased across most biofilm layers (*P* ≤ 0.043-0.0012). Controls had significantly lower counts of *Gardnerella* spp. and *F. vaginae. P. bivia* had low counts in all specimens. During the critical time period surrounding iBV, *Gardnerella* spp. are abundant throughout the developing biofilm and facilitate *F. vaginae* incorporation at later time points and higher biofilm layers. Additional research, including other *Prevotella* spp., is needed.

**IMPORTANCE:**

Bacterial vaginosis (BV) is the most common vaginal infection in reproductive-age women worldwide with a global prevalence of 30%. Recurrence rates can be up to 60% within 1 year of treatment. While BV is characterized as a polymicrobial biofilm infection, the exact etiology remains unknown. The BV biofilm may persist after antibiotic treatment, possibly due to incomplete eradication by current antimicrobial therapies, contributing to recurrent infection. Data are limited in evaluating the spatial formation of the BV biofilm around the time of incident BV. Providing a better understanding of this critical time period in incident BV pathogenesis is necessary to inform the development of prevention methods aimed at inhibiting biofilm formation and improving long-term treatment outcomes.

## INTRODUCTION

Bacterial vaginosis (BV) is the most common vaginal infection worldwide, with recurrence rates up to 60% within one year of antimicrobial therapy (1). BV is associated with an increased risk of infertility, adverse birth outcomes, post-operative gynecologic infections, HIV/sexually transmitted infection (STI) acquisition, and pelvic inflammatory disease (2). It is estimated to account for $4.8 billion USD in global treatment costs annually (1). BV is characterized by loss of protective vaginal *Lactobacillus* spp., such as *Lactobacillus crispatus*, and overgrowth of facultative and strict anaerobic bacteria including *Gardnerella* spp., *Prevotella bivia*, and *Fannyhessea vaginae* (3, 4). BV-associated bacteria (BVAB) adhere to vaginal epithelial cells (VECs), forming a polymicrobial biofilm (5). Maturation of the BV biofilm induces epithelial cell shedding and results in the formation of the characteristic “clue cell” seen on wet mount microscopy (6, 7). The mechanisms governing BV biofilm formation, particularly the processes that initiate its development and the dynamics of bacterial colonization, are not fully understood (6). Because of this knowledge gap, the etiology of BV remains unclear. A better understanding of this critical time period in incident BV (iBV) pathogenesis is necessary to inform the development of prevention methods aimed at inhibiting biofilm formation and improving long-term treatment outcomes.

We have previously developed a conceptual model of BV biofilm formation in which *Gardnerella* spp. and *P. bivia* are early colonizers that engage in a synergistic relationship through the exchange of metabolites (4, 8). In our model, *Gardnerella* spp. tolerate the high-redox potential of a *Lactobacillus*-dominant vaginal community as facultative anaerobes capable of growth under both aerobic and anaerobic conditions (9), serving as the primary initiators of the BV biofilm (4, 8). *P. bivia* then joins the biofilm by utilizing amino acids produced by *Gardnerella* spp. and, in turn, produces ammonia (10). The ammonia produced by *P. bivia* enhances *Gardnerella* spp. growth, promoting further biofilm development (10). *F. vaginae* (and other BVAB) are thought to be secondary colonizers in the setting of mucin degradation caused by the virulence factors of *Gardnerella* spp. and *P. bivia* (4).

In our recent longitudinal study of heterosexual women who developed iBV (cases) compared to women who did not develop iBV (controls) (11), we demonstrated that *Gardnerella* spp. counts were significantly higher in iBV cases compared to controls 5 days pre-iBV whereas *F. vaginae* counts were significantly higher in cases compared to controls on the day of iBV diagnosis (10). *P. bivia* counts were low in all of the specimens analyzed as part of this cohort (11). These previous results support the majority of our conceptual model (4, 8), suggesting that *Gardnerella* spp. are early colonizers of the BV biofilm while *F. vaginae* is a secondary colonizer (11). However, given the low counts of *P. bivia* in all specimens analyzed, the role of other *Prevotella* spp. beyond *P. bivia* should be explored.

As an extension of this recent work (11), we aimed to explore the longitudinal spatial organization of *Gardnerella* spp., *P. bivia*, and *F. vaginae* among the layers of the BV biofilm over time utilizing peptide nucleic acid-fluorescence *in situ* hybridization (PNA-FISH) in our iBV vaginal specimens and matched control specimens (12). In concordance with our BV model (4, 8), we hypothesized that, among women who developed iBV, *Gardnerella* spp. would form the lower layers of the BV biofilm while *F. vaginae* would form the upper layers. Although we originally hypothesized that *P. bivia* would join the lower layers of the biofilm due to synergy with *Gardnerella* spp., based upon our recent findings using the same cohort (11), we did not expect to find a significant amount of *P. bivia* in our specimens in this study.

## MATERIALS AND METHODS

### Enrollment and vaginal specimen collection

Enrollment for this study was from November 2020 to August 2024 in the Birmingham, Alabama Metropolitan area. Previous methods for study enrollment, inclusion/exclusion criteria, and specimen collection have been published (11, 12). Potential participants attended a study screening visit at the University of Alabama at Birmingham (UAB) Sexual Health Research Clinic (SHRC) and completed a study survey (Table S1). Women were evaluated for signs of vaginal infection, tested for BV by the Amsel criteria (13) and Nugent score (14), and tested for *Chlamydia trachomatis*, *Neisseria gonorrhoeae*, *Trichomonas vaginalis*, and *Mycoplasma genitalium* using the Roche Cobas 6800 CT/GC and TV/MG assays (15, 16). Enrolled women without any active vaginal infection(s) and/or STI(s) were taught to self-collect three vaginal swab specimens twice daily for 60 days, smearing the first specimen on a glass slide for subsequent Nugent score determination in our research laboratory. They also completed daily diaries providing information about sexual activity, menses, genital symptoms, and medication use (Table S1). Aliquots of one of the three vaginal swab specimens were used for the purposes of this study.

Self-collected specimens were refrigerated at the participants’ homes until weekly drop-off at the clinical site. After drop-off, vaginal specimens were aliquoted into cryo-storage tubes and stored at −80°C in our research laboratory. Data from each participant, including socio-demographics, STI history, sexual behavior data, and Nugent scores, were stored in a REDCap database (17).

### Selection of vaginal specimens for PNA-FISH

**Fig 1:**
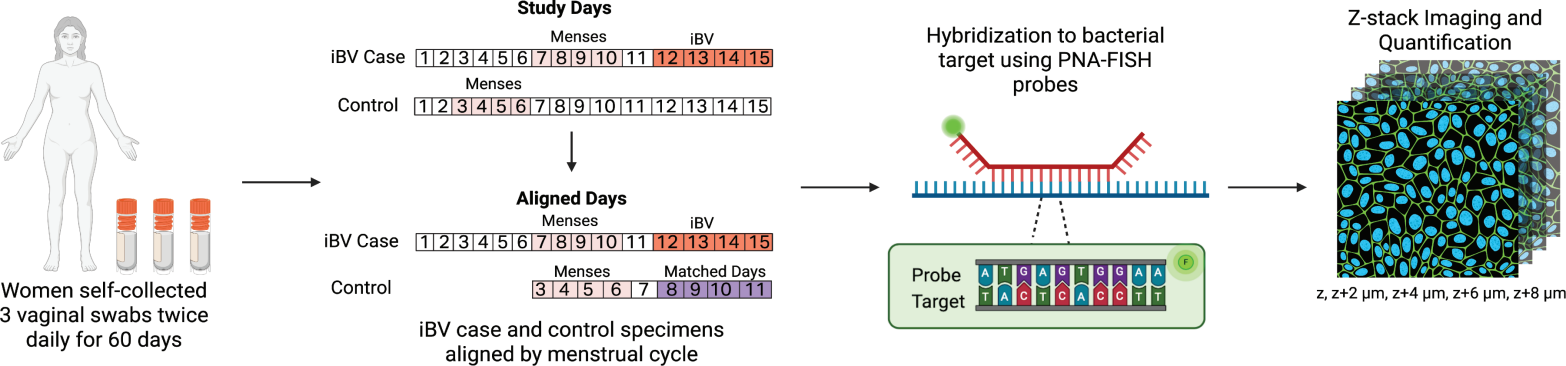
Experimental methods to determine bacterial counts among the layers of the BV biofilm. Vaginal specimens were collected using nylon-tipped flocked swabs and placed in separate tubes with 1 mL of a 1:10 dilution of AssayAssure and 1× phosphate-buffered saline (PBS). iBV case specimens were aligned to matched control specimens by day of menses (represented by pink segment). Selected days for study procedures are shown in red (cases) or purple (controls) segments.

iBV was determined by a Nugent score of 7–10 on ≥4 consecutive vaginal specimens during the course of the study (14). Women who were diagnosed with iBV (i.e., cases) were matched in a 1:1 fashion to women who did not develop iBV (i.e., healthy controls) by age (±5 years), self-identified race, and contraceptive method (12). Participants who maintained a normal Nugent score of 0–3 for the majority (≥85%) of study days were eligible to be selected as a healthy control. For the purposes of this study, vaginal specimens from 2 days pre-iBV diagnosis, the day of iBV diagnosis, and 2 days post-iBV diagnosis were selected from each iBV case participant for bacterial biofilm layer quantification. These time points were selected based on data obtained in our previous iBV pathogenesis study which found that the mean relative abundance of *G. vaginalis* became significantly higher 3 days prior to iBV while *F. vaginae* relative abundance became significantly higher on the day of iBV (3). We anticipated that selection of these time points would enable us to capture short-term changes in the counts and spatial organization of our bacterial species of interest immediately before, during, and after the onset of iBV. Specimens from healthy controls were imaged on the same days, aligned by menstrual cycle day, as their corresponding iBV case participant (Fig. 1).

### PNA-FISH fixation and hybridization

Methods for PNA-FISH fixation and hybridization have been previously published (5, 11, 18–21). Briefly, vaginal specimens were thawed and gently vortexed to suspend bacteria and VECs. A total of 20 µL of each vaginal specimen from 2 days pre-iBV, the day of iBV, and 2 days post-iBV diagnosis was fixed onto glass slides at 37°C. Each fixed vaginal specimen was then incubated for 90 min at 60°C with 20 µL of hybridization solution containing 200 nM of the PNA probes specific for *Gardnerella* spp. 16S rRNA (originally developed as a *G. vaginalis* probe prior to the taxonomic identification of other *Gardnerella* spp.) (18), *P. bivia* 23S rRNA (19), and *F. vaginae* 23S rRNA (20) (PNA Bio). To detect the presence of bacterial DNA and the nuclei of VECs, counterstaining was performed on the hybridized specimens with 1:1,000 4′,6′-diamidino-2-phenylindole (DAPI) diluted in 1× PBS. The PNA probes used in this study have been fully optimized and used in several other published studies (11, 18–20).

### Fluorescent imaging

**Fig 2:**
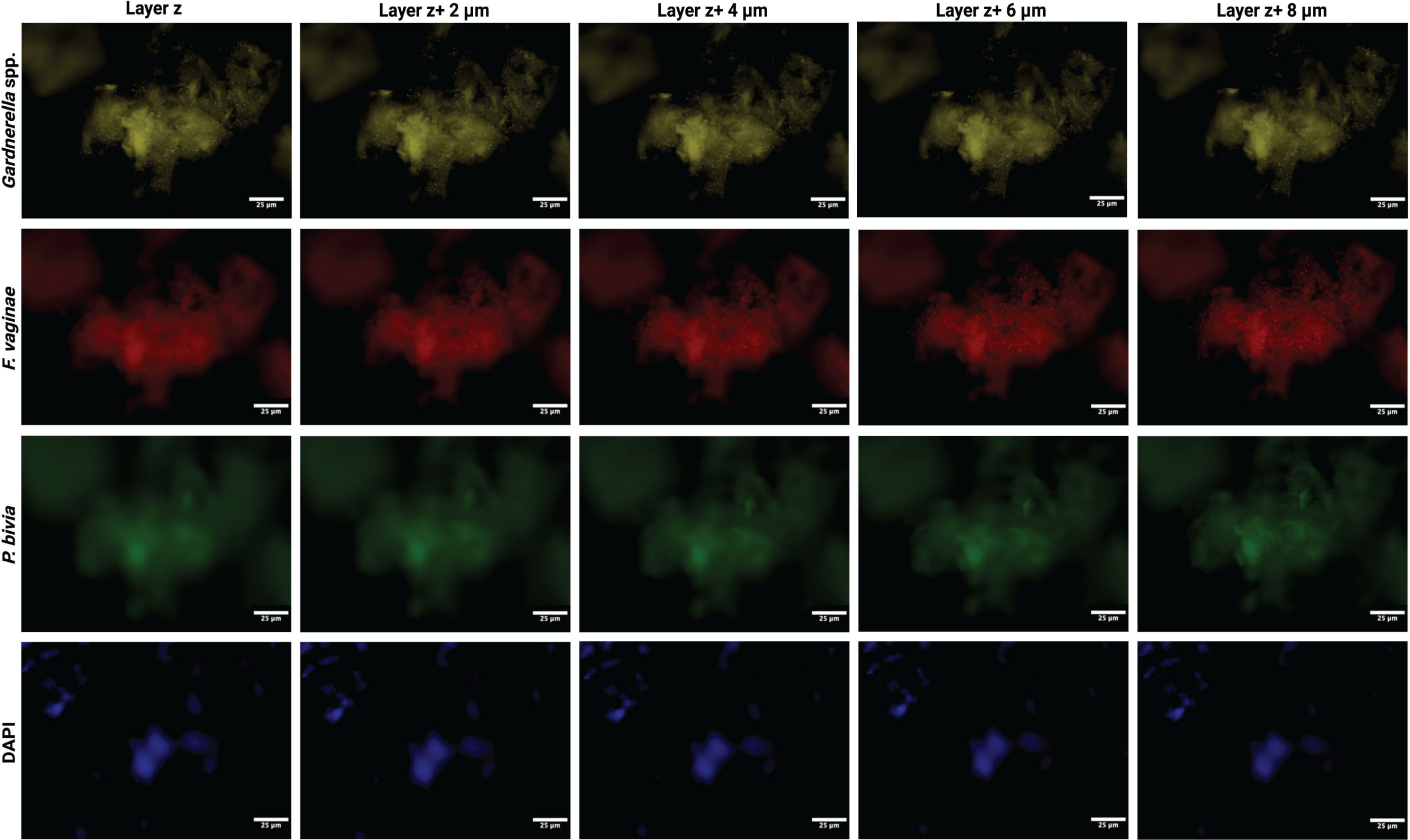
Separated channel view of a biofilm 2 days post-iBV from one iBV case participant. Images taken at layers *z*, *z* + 2, *z* + 4, *z* + 6, and *z* + 8 μm, at 80× magnification on the NanoZoomer S60 Slide Scanner. *Gardnerella* spp. are yellow, *F. vaginae* is red, *P. bivia* is green, and DAPI is blue. Layer *z* represents the bottom layer of the BV biofilm.

Three fluorescent images per iBV specimen were captured (22) using the NanoZoomer S60 Slide Scanner (Hamamatsu Corporation) Z-stack function at biofilm optical layers *z* (basal), *z* + 2, *z* + 4, *z* + 6, and *z* + 8 μm (apical). Single bacterial cells dispersed irregularly across a specimen were not considered a biofilm. Aggregates of bacteria adherent to VECs and composed of 1 or more bacterial species were defined as a biofilm. To normalize biofilm size across images, only distinct biofilms not attached to larger conglomerates of bacteria and VECs were imaged, ensuring that the complete biofilm could be visualized at 80× magnification (Fig. S1). Layer *z* was determined as the layer in which the VECs were in focus on the slide scanner. The scanner identifies the focal midpoint (i.e., layer *z* + 4 μm) first, proceeds downward, and then upwards through the Z-stack. *Gardnerella* spp., *P. bivia*, and *F. vaginae* were viewed under an 80× objective via their respective PNA probe through the TRITC, FITC, and CY5 filters, respectively (Fig. 2). For each slide, exposure settings were optimized and held constant, preventing differences due to wavelength. Following image capture, counts of each of the bacterial species of interest per layer were recorded using Fiji ImageJ 1.8.0 (11). To ensure accurate quantification, each bacterial species of interest was quantified separately using its species-specific fluorescent filter. Contrast thresholds were optimized to distinguish bacterial cells from background signal. Touching bacterial cells were separated by the “watershed tool” to predict the separation between different cells. Then, the software provided the bacterial cell count for each biofilm layer using the “analyze particles” feature. For specimens from each case and control, bacterial counts were averaged for each of the five biofilm layers across the three replicate biofilms. These layer and bacteria-specific means were then pooled with the corresponding layer and bacteria means from other iBV case biofilms.

### Statistical analysis

All statistical analyses were performed using GraphPad Prism 10.0.2. The Wilcoxon matched-pairs signed-rank test was used to compare pooled mean bacterial counts between *Gardnerella* spp., *P. bivia*, and *F. vaginae* across days and between cases and controls due to the non-parametric and paired nature of the count data. Significance of the total bacterial counts of analyzed bacterial spp. on the different time points was determined by a Friedman test with a Dunn’s post hoc test. A repeated measures ANOVA with the Tukey’s post-hoc test was used to evaluate significance of both *Gardnerella* spp. and *F. vaginae* across biofilm layers. *P*-values ≤0.05 were deemed statistically significant.

## RESULTS

**Fig 3:**
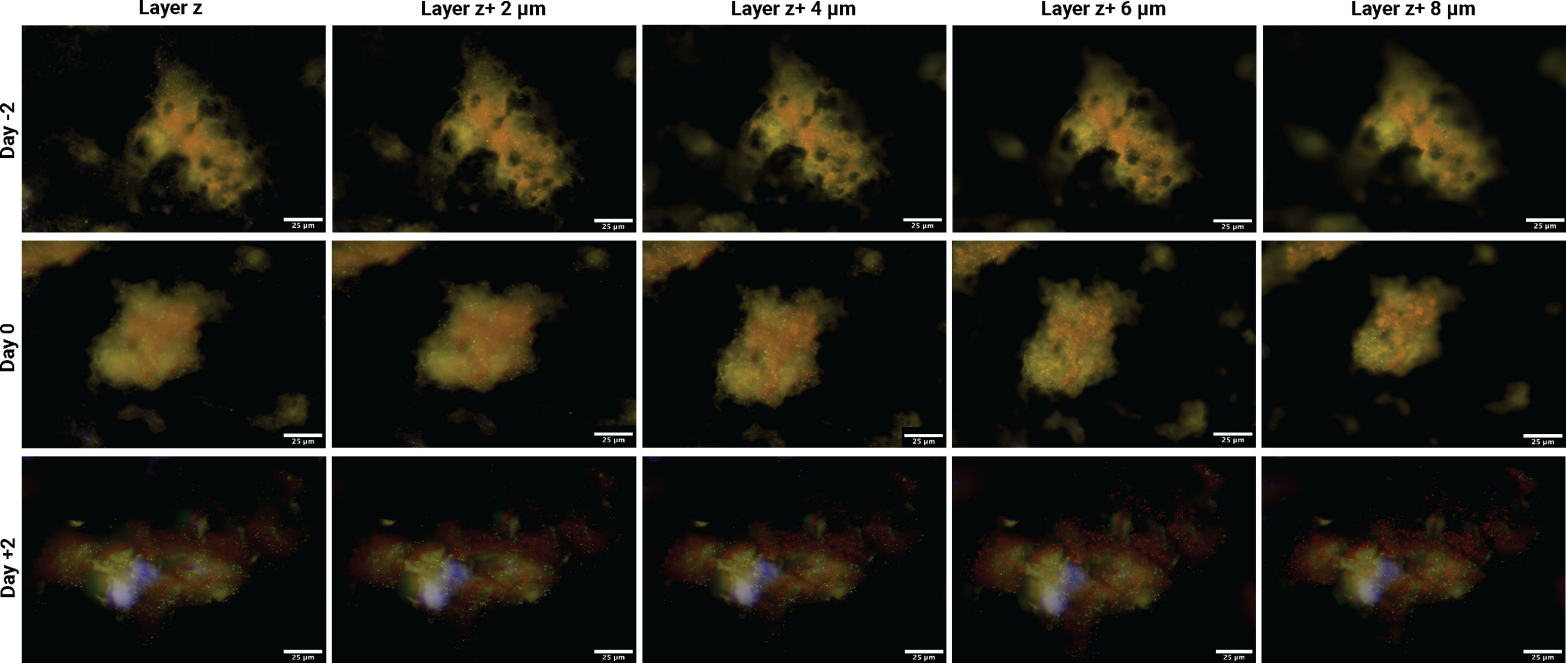
Representative images taken on different BV biofilm layers of one iBV case 2 days pre-iBV (Day −2), day of iBV (Day 0), and 2 days post-iBV diagnosis (Day +2). Images taken at layers *z*, *z* + 2, *z* + 4, *z* + 6, and *z* + 8 μm, at 80× magnification on the NanoZoomer S60 Slide Scanner. *Gardnerella* spp. are in yellow, *F. vaginae* is in red, and DAPI is blue. Layer *z* represents the bottom layer of the BV biofilm.

A total of 108 vaginal specimens (54 iBV case specimens and 54 control specimens) from 18 iBV case and 18 control participants were analyzed in this study. There were no significant differences in the characteristics (i.e. age, race, ethnicity, education, STI history, BV history, and contraception history) between the iBV case and control participants, as previously described (11). Specimens from 2 days pre-iBV (Day −2), day of iBV (Day 0), and 2 days post-iBV (Day 2) were fluorescently imaged at five optical biofilm layers, *z* (basal layer), *z* + 2, *z* + 4, *z* + 6, and *z* + 8 μm (apical layer), to quantify the bacterial spatial organization of *Gardnerella* spp., *P. bivia*, and *F. vaginae* in each specimen (Fig. 3; Fig. S2). For each iBV case and control specimen, three representative fields of view were imaged (22), totaling 324 captured images across all specimens for analysis.

**Fig 4:**
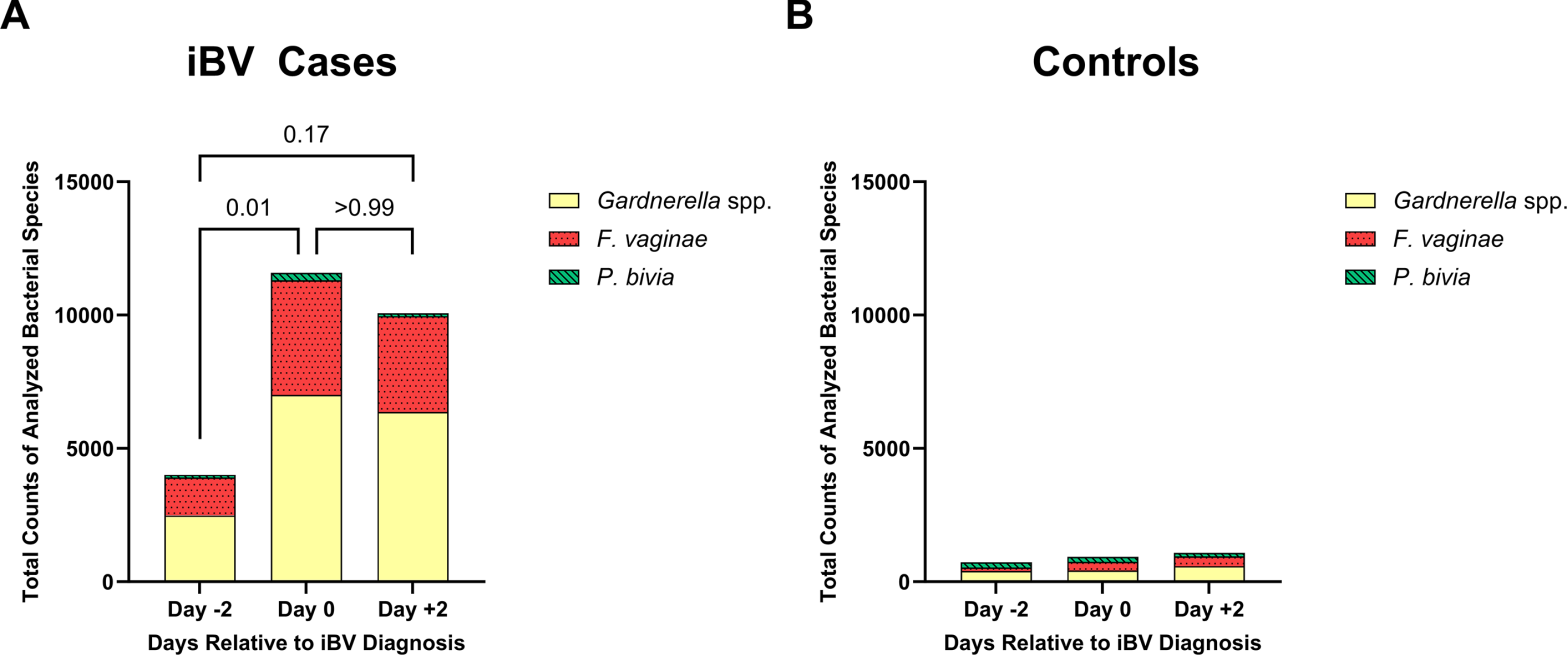
Total pooled bacterial counts of *Gardnerella* spp. (yellow), *F. vaginae* (red, dotted), and *P. bivia* (green, striped) in iBV cases and controls. (**A**) Total pooled bacterial counts in BV biofilms 2 days pre-iBV (Day −2), day of iBV (Day 0), and 2 days post-iBV diagnosis (Day +2). *P* values were determined by a Friedman test with a Dunn’s post hoc test. (**B**) Total pooled bacterial counts in control specimens 2 days pre-iBV (Day −2), day of iBV (Day 0), and 2 days post-iBV diagnosis (Day +2).

Total pooled bacterial counts of *Gardnerella* spp., *P. bivia*, and *F. vaginae* were significantly higher on the day of iBV compared to 2 days pre-iBV (*P* = 0.011) (Fig. 4A). Total pooled counts of all three bacteria on the day of iBV and 2 days post-iBV were similar (*P* > 0.99) (Fig. 4A). No significant difference in total pooled bacterial counts of *Gardnerella* spp., *P. bivia*, and *F. vaginae* was observed between 2 days pre-iBV and 2 days post-iBV (*P* = 0.17); however, total pooled bacterial counts 2 days post-iBV remained elevated compared to the day of iBV. *P. bivia* detection among all cases was limited (<10 cells) across the layers of all biofilms imaged on all days (Fig. 4A), as previously noted (11). In contrast to iBV case participants, total pooled bacterial counts of *Gardnerella* spp., *P. bivia*, and *F. vaginae* were very low across all controls (Fig. 4B). Due to the absence of biofilm structures, control specimens had significantly lower pooled mean bacterial counts of *Gardnerella* spp. and *F. vaginae* compared to iBV case specimens, although all control specimens had detectable, but very low levels of both species (Fig. S3). There were no significant differences between the pooled mean counts of *P. bivia* between cases and controls (Fig. S3).

**Fig 5:**
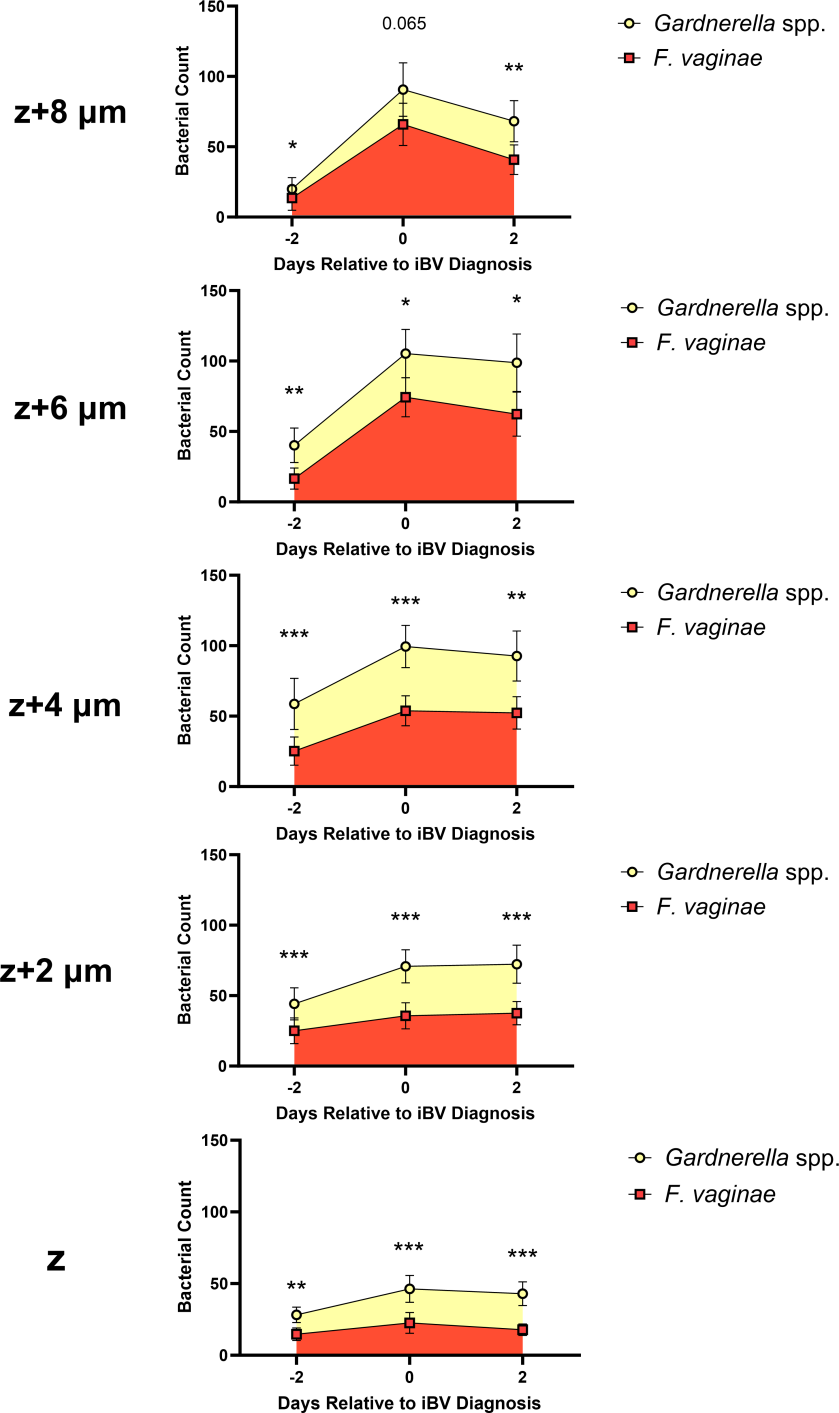
Pooled counts of *Gardnerella* spp. (yellow) and *F. vaginae* (red) on biofilm layers (*z*, *z* + 2, *z* + 4, *z* + 6, and *z* + 8 μm) across 2 days pre-iBV (Day −2), day of iBV (Day 0), and 2 days post-iBV (Day +2) in specimens from participants with iBV. The Wilcoxon matched pairs signed rank test was used to compare species counts (*P ≤ 0.05, **P ≤ 0.01, and ***P ≤ 0.001). Error bars represent the standard error of the mean. Layer *z* represents the bottom layer of the BV biofilm.

Across all days and biofilm layers imaged, pooled mean *Gardnerella* spp. counts were significantly higher than pooled mean *F. vaginae* counts (*P* ≤ 0.022–0.0003), except for layer *z* + 8 μm on the day of iBV (*P* = 0.065), where the bacterial counts were similar (Fig. 5). Reduced mean bacterial counts of both *Gardnerella* spp. and *F. vaginae* were noted in the upper biofilm layers 2 days pre-iBV, suggesting that the biofilm was in an early developmental stage at this time (Fig. 5). On day 2 pre-iBV*,* pooled mean *F. vaginae* counts were low across all layers, indicating minimal colonization prior to iBV (Fig. 5). On the day of iBV and day 2 post-iBV, *F. vaginae* and *Gardnerella* spp. displayed similar counts in the top layers of the biofilm (Fig. 5). Interestingly, both *Gardnerella* spp. and *F. vaginae* pooled mean counts peaked on the day of iBV and then slightly decreased 2 days post-iBV, which may reflect the beginning of bacterial growth stabilization (Fig. 5). Although individual iBV case participants exhibited some variability in total bacterial counts, participant-level data demonstrated that these same temporal trends were consistent across most individual participants (Fig. S4).

**Fig 6:**
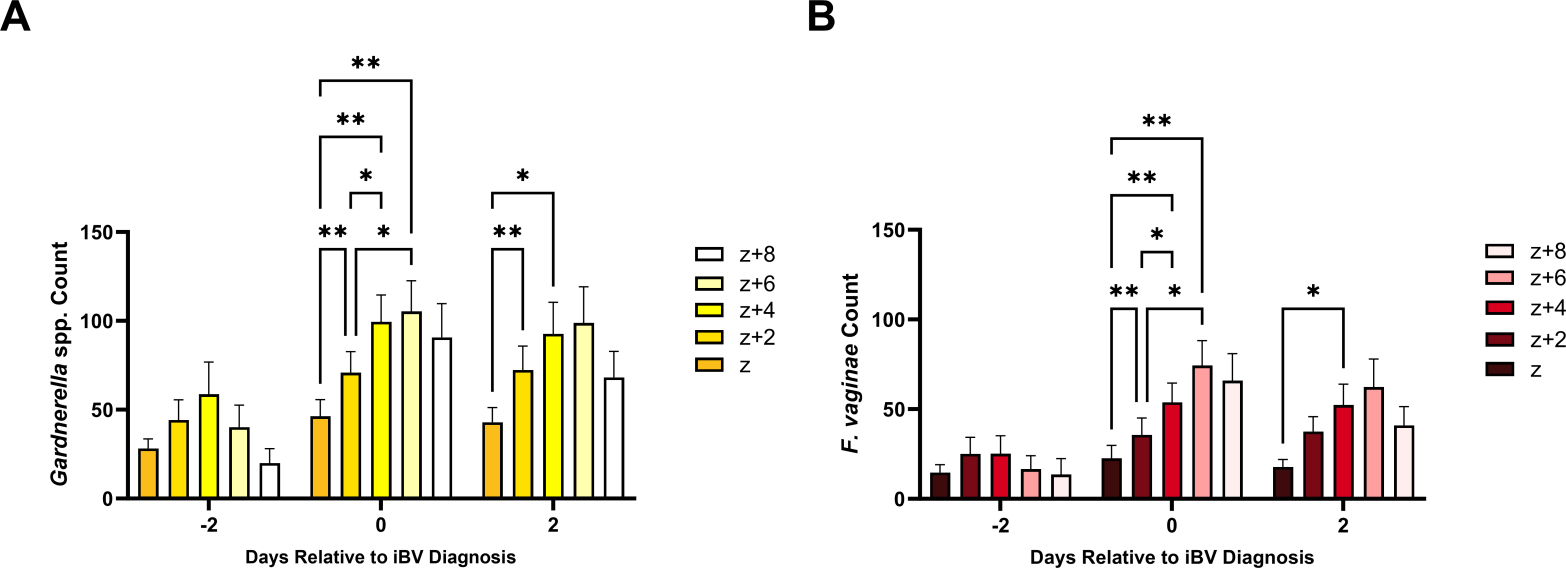
Pooled counts of *Gardnerella* spp. (yellow) (**A**) and *F. vaginae* (red) (**B**) compared across the layers of BV biofilms (z, z + 2, *z* + 4, *z* + 6, and *z* + 8 μm) 2 days pre-iBV (Day −2), day of iBV (Day 0), and 2 days post-iBV (Day +2). The repeated measures ANOVA with the Tukey’s post hoc test was used to compare species counts across biofilm layers (*P ≤ 0.05 and **P ≤ 0.01). Error bars represent the standard error of the mean. Layer *z* represents the bottom layer of the BV biofilm.

We subsequently analyzed the pooled mean counts of *Gardnerella* spp. and *F. vaginae* across the different layers of the BV biofilm during the three time points of interest. On day 2 pre-iBV, the pooled mean counts of both *Gardnerella* spp. and *F. vaginae* in all biofilm layers did not significantly differ (Fig. 6). On the day of iBV, mean pooled counts of both *Gardnerella* spp. and *F. vaginae* displayed a significant sequential increase among all layers of the biofilm (*P* ≤ 0.043–0.0012), with the highest counts in the upper layers of the biofilm (Fig. 6). Pooled mean *Gardnerella* spp. counts were significantly higher on layer *z* + 2 μm compared to layer *z* (*P* = 0.022), and layer *z* + 4 μm compared to layer *z* (*P* = 0.0056) on day 2 post-iBV. Pooled mean *F. vaginae* counts were significantly higher on layer *z* + 4 μm compared to layer *z* (*P* = 0.040) on day 2 post-iBV.

## DISCUSSION

Multiple studies have identified that BV is a polymicrobial biofilm infection (23–27); however, little work has been done to understand the spatial organization of key BVAB within the biofilm immediately preceding, during, and after iBV diagnosis. To our knowledge, this study is the first to longitudinally analyze the spatial organization of our key BVAB of interest, *Gardnerella* spp., *P. bivia*, and *F. vaginae*, within the layers of the developing BV biofilm. In this study, we utilized PNA-FISH and novel digital slide-scanning imaging techniques in order to accomplish our objective. Previous work has demonstrated key BVABs in the biofilm among vaginal specimens (5, 21, 28); however, this work was cross-sectional and did not capture the temporal or spatial dynamics of biofilm development. Spatial arrangement and temporal dynamics are critical factors in bacterial interactions, influencing metabolite exchange, virulence factor activity, and resistance to host defenses and antibiotic treatment (29). Elucidating the spatial and temporal organization provides critical insight into BV biofilm formation, advancing our understanding of iBV pathogenesis.

In contrast to our hypothesis, pooled mean *Gardnerella* spp. counts were significantly higher than pooled mean *F. vaginae* counts across most layers and time points within the BV biofilm in this study. We also observed a significant spatial distribution trend in which both *Gardnerella* spp. and *F. vaginae* were increasingly present in the upper layers of the biofilm on the day of iBV diagnosis. Individual participant total bacterial count data also supported this pattern in most iBV cases. Consistent with our current hypothetical model (4, 8), pooled mean *F. vaginae* counts increased in the upper layers of the biofilm, reaching levels comparable to *Gardnerella* spp. counts in layers *z* + 6 and *z* + 8 μm starting on the day of iBV. However, *F. vaginae* counts never exceeded *Gardnerella* spp. counts at any point in this study, contrary to our hypothesis. Thus, these findings refine our iBV pathogenesis conceptual model (4, 8): *Gardnerella* spp. initiate BV biofilm formation, but are distributed throughout the layers of the biofilm rather than restricted to the basal layers. The *Gardnerella*-dominated biofilm subsequently facilitates the attachment of secondary colonizers, such as *F. vaginae*, starting on the day of iBV.

Consistent with these results, Swidsinski et al. observed persistent BV biofilms primarily composed of *G. vaginalis* and *F. vaginae* in women after treatment with oral metronidazole (30). Additionally, several studies have indicated that *F. vaginae* abundance depends on the presence of *Gardnerella* spp. (31–33) and their common co-occurrence in the BV biofilm (5). Taken together, these results confirm that *Gardnerella* spp. and *F. vaginae* are key constituents of the BV biofilm, display spatial organization and co-localization within the BV biofilm, and warrant greater focus to prevent BV biofilm formation and infection persistence.

*P. bivia* counts were overall very low within the BV biofilm, despite prior studies supporting its involvement in iBV pathogenesis (25, 27, 34). However, the methods used in these previous studies were different from the methods used here, which could have accounted for different results. We expected this outcome based on our previous findings of low *P. bivia* counts in these specimens using PNA-FISH (11). One possible explanation for this finding is that other *Prevotella* spp. may be more involved in BV pathogenesis than *P. bivia*. This could include *P. timonensis,* which has recently been implicated to have greater sialidase production compared to *Gardnerella* spp. and other *Prevotella* spp. (35–37). We are currently developing a new *Prevotella* spp. PNA probe to further explore this question.

Our results also demonstrate that the BV biofilm is within its early formation stages on day 2 pre-iBV, as evidenced by significantly lower bacterial counts compared to the day of iBV. These data suggest that BV biofilm formation begins before BV can be clinically diagnosed, allowing the polymicrobial biofilm to undergo initial establishment. Thus, the early establishment of the BV biofilm highlights a critical stage in iBV pathogenesis that precedes the current diagnostic threshold of the Nugent scoring method. While the Nugent score is not typically used in a clinical setting, it has been traditionally regarded as the gold standard for BV diagnosis, prior to the availability of BV molecular diagnostic tests (38, 39). Failure to adequately identify and treat the BV biofilm at this early stage allows progression of infection in women.

Our findings highlight the need for methods that simultaneously target multiple BVAB and disrupt the biofilm structure to improve long-term treatment outcomes (40). Combining antimicrobial therapy with biofilm-disrupting agents may be beneficial for women with recurrent or persistent BV (41); this will need to be investigated in future studies. Additionally, a better understanding of the spatial organization of the BV biofilm could provide insights into antimicrobial penetration into the biofilm and inform effective disruption methods. Ultimately, implementing biofilm-targeted treatment strategies has the potential to further reduce recurrence rates and improve long-term clinical outcomes.

Limitations of this study include the participants’ self-collected and self-stored vaginal specimens, which could have influenced biofilm preservation and bacterial counts. However, our team maintained regular contact with participants to ensure correct specimen collection and storage (12). Self-collected specimen methods are commonly used in many studies (5, 12, 18, 21, 42) to capture longitudinal trends in the same participant as it is not feasible or practical to collect other types of vaginal specimens on a daily basis, such as vaginal biopsies. Therefore, our analysis is on sloughed epithelial cells and not directly on the vaginal epithelium. Second, the vaginal specimens in this study underwent freezing, thawing, and gentle vortexing for PNA-FISH methods, which could have affected the quality of the specimens and disrupted parts of the biofilm. Thus, the biofilms that we describe in this study may have been influenced by variables introduced during sample processing. Third, we only visualized three key BVAB, *Gardnerella* spp., *P. bivia*, and *F. vaginae*, in this study based on the current ability of our PNA probes. Fourth, optical sections were acquired at 2 μm intervals, and it is possible that a single bacterial cell could have generated fluorescent signal in more than one *z*-layer. However, only in-focus cells with high fluorescent intensity were counted. Any potential overcounting would have been systematic and non-differential between images. Additionally, across specimens, bacterial counts were consistently the lowest on layer *z*. In wide-field fluorescence systems, such as the NanoZoomer S60 slide scanner used in this study, deeper planes can experience reduced signals, which may have contributed to this result (43). Lower count patterns could have also occurred due to technical or biological factors (44). Fifth, Nugent scoring was performed to diagnose iBV in this study instead of a molecular diagnostic assay for multiple reasons. First, it would have been cost-prohibitive to perform this assay on the large number of vaginal specimens collected for the duration of this study. Second, BV molecular diagnostic assays are currently only FDA-approved for use in symptomatic women; women in our study were asymptomatic at baseline and not all developed symptoms during the study (data not shown). Additionally, while we previously observed that the abundance of *Gardnerella* spp. significantly increases in iBV cases 4–5 days pre-iBV (3, 11), we chose to focus on 2 days pre-iBV to 2 days post-iBV in this study to focus on early interactions between our bacterial species of interest in the early development stages of the BV biofilm before, during, and after iBV onset. Finally, only distinct biofilms fully imaged at 80× magnification were considered due to normalization between specimens, potentially underrepresenting larger or irregularly shaped biofilms. However, using this approach, we were able to directly compare *Gardnerella* spp., *P. bivia*, and *F. vaginae* on the *ex vivo* BV biofilms in our study.

In summary, this study provides novel insight into the longitudinal and spatial organization of several key BVAB within the layers of the BV biofilm. Our findings indicate that BV biofilm formation begins at least 2 days before iBV diagnosis and begins to stabilize by 2 days post-iBV, highlighting a critical window in iBV pathogenesis. *F. vaginae* counts become comparable to *Gardnerella* spp. counts toward the upper layers of the biofilm. Both *Gardnerella* spp. and *F. vaginae* counts were significantly higher on the upper layers of the biofilm. Future work should expand this analysis to a larger cohort of women and analyze additional BVAB within the biofilm, such as the individual species of the *Gardnerella* genus, *Prevotella* spp. other than *P. bivia*, *Sneathia* spp., and *Megasphaera* spp. (4, 8).

## SUPPLEMENTAL MATERIAL

Supplemental material iai.00630-25-s0001.pdf Fig. S1 to S4; Table S1.

ASM does not own the copyrights to Supplemental Material that may be linked to, or accessed through, an article. The authors have granted ASM a non-exclusive, world-wide license to publish the Supplemental Material files. Please contact the corresponding author directly for reuse.
