# Supplementary material for: Spatial organization of Gardnerella species, Prevotella bivia, and Fannyhessea vaginae in the bacterial vaginosis biofilm
Source: Infect Immun. 2026 Jan 22;94(2):e00630-25. doi: 10.1128/iai.00630-25 (PMC12890030; doi:10.1128/iai.00630-25)
Supplement: Supplemental material — Fig. S1 to S4; Table S1. [file iai.00630-25-s0001.pdf]

Supplemental Material

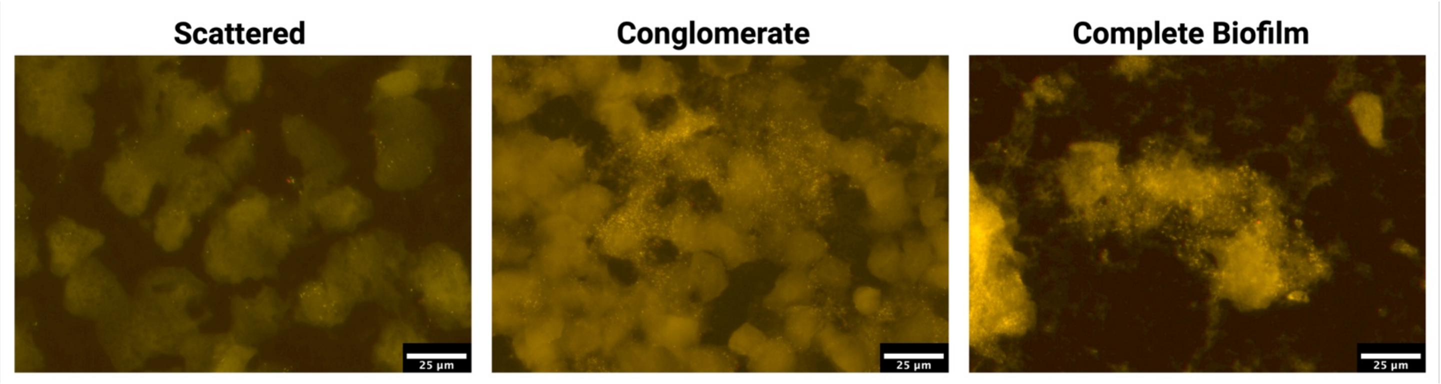

Supplemental Figure 1. Examples of scattered bacteria across vaginal epithelial cells, large biofilm conglomerates, and a complete biofilm at 80X magnification on the NanoZoomer S60 Slide Scanner. *Gardnerella* spp. are yellow and *F. vaginae* is red.

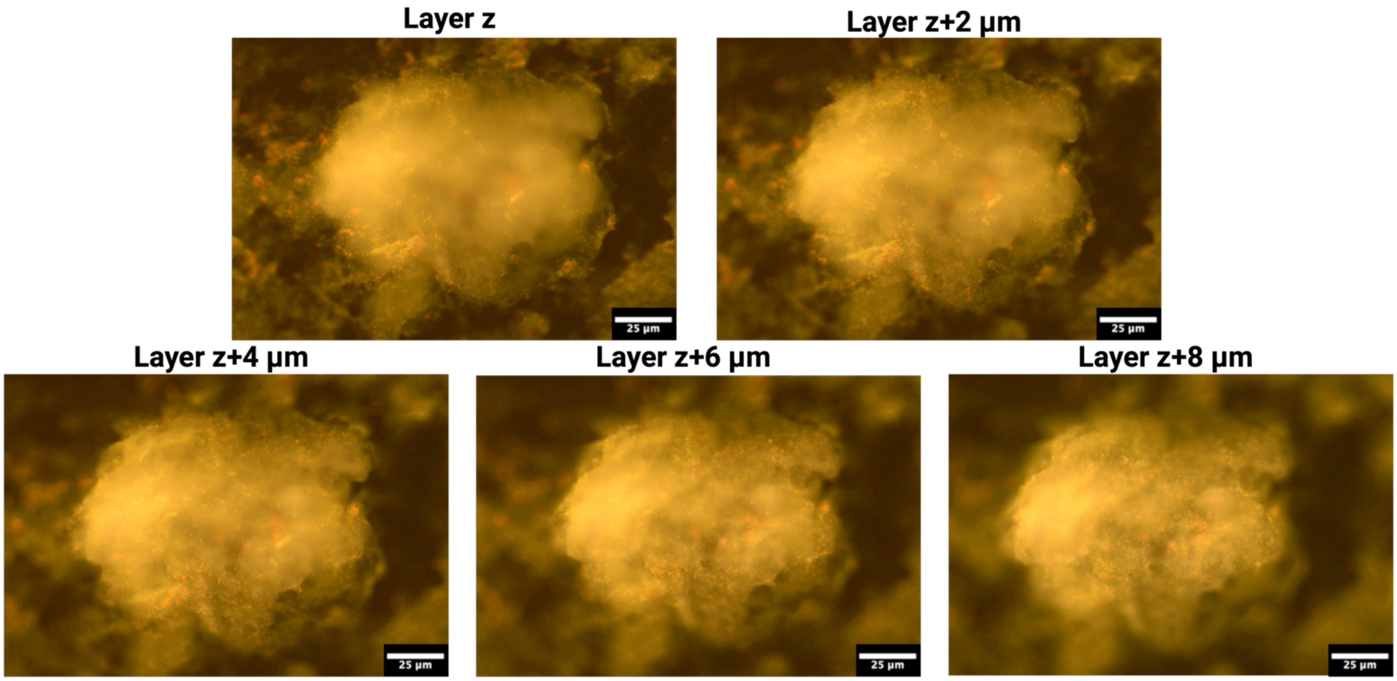

Supplemental Figure 2. Close-up images of a biofilm 2 days post-iBV taken at layers z, z+2 µm, z+4 µm, z+6 µm, and z+8 µm at 80X magnification on the NanoZoomer S60 Slide Scanner. *Gardnerella* spp. are yellow and *F. vaginae* is red. Layer z represents the bottom of the BV biofilm.

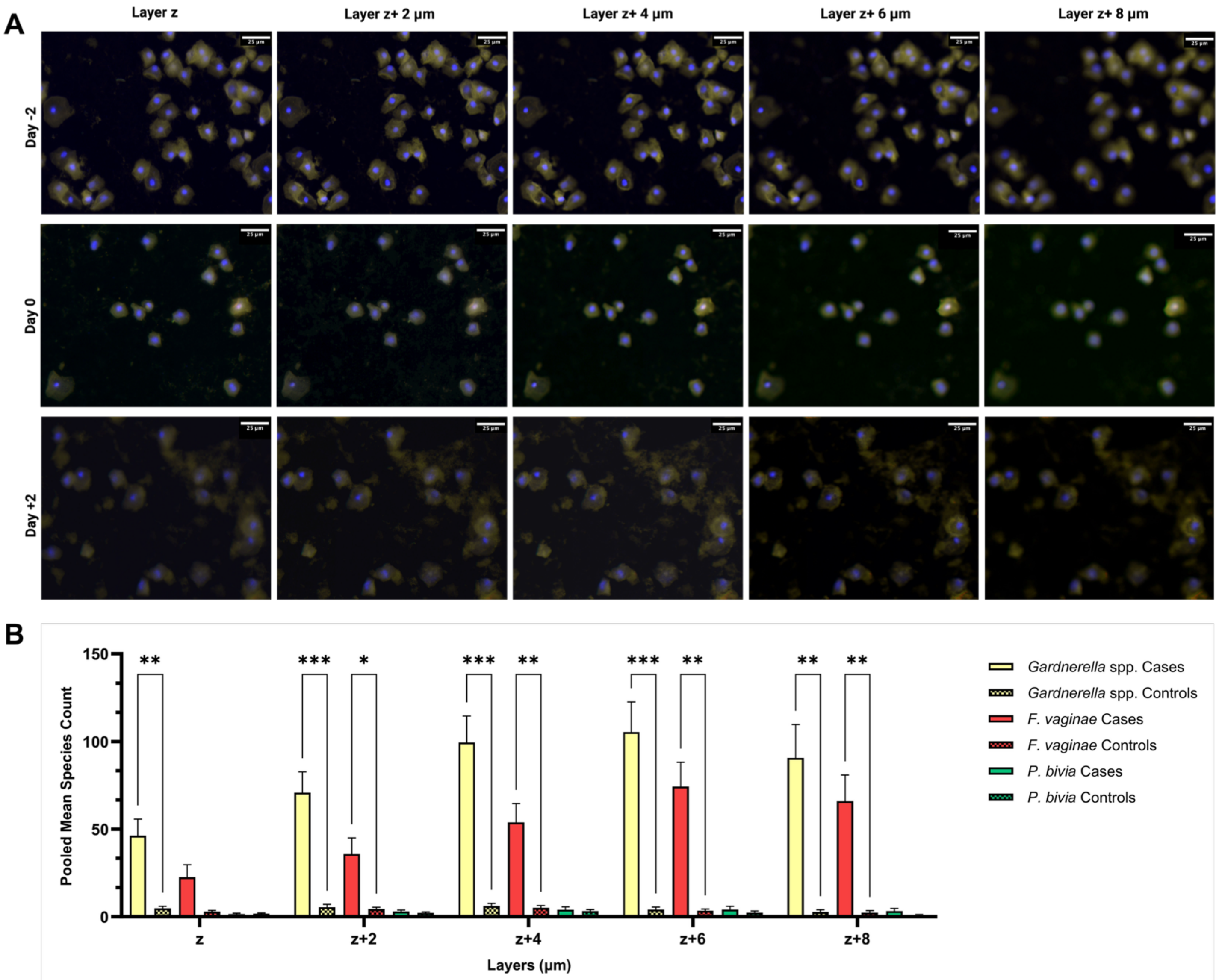

Supplemental Figure 3. Control images and pooled mean bacterial counts compared between iBV cases and controls. A) Control representative images captured on the matched day of 2 days pre-iBV (Day -2), day of iBV (Day 0), and 2 days post-iBV diagnosis (Day +2). Images taken at layers z, z+2 μm, z+4 μm, z+6 μm, and z+8 μm at 80X magnification on the NanoZoomer S60 Slide Scanner. *Gardnerella* spp. are yellow, *F. vaginae* is red, *P. bivia* is green, and DAPI is blue. Layer z represents the bottom layer of the BV biofilm. B) Control pooled mean bacterial counts compared to pooled mean iBV case counts on the day of iBV. The Wilcoxon matched pairs signed rank test was used to compare species counts between cases and controls (*$p \leq 0.05$, **$p \leq 0.01$, ***$p \leq 0.001$). Error bars represent the standard error of the mean.

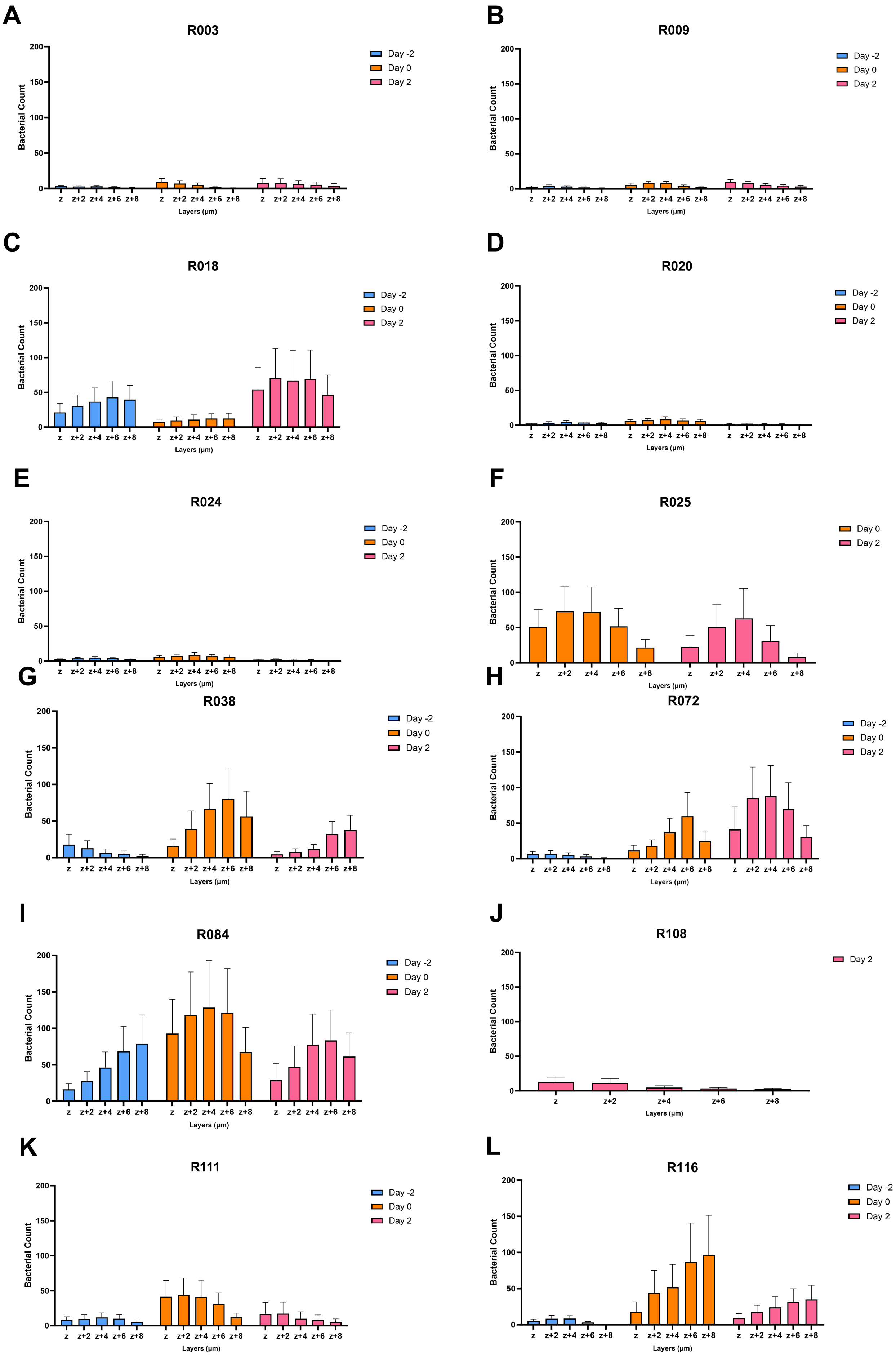
A
R003
B
R009
C
R018
D
R020
E
R024
F
R025
G
R038
H
R072
I
R084
J
R108
K
R111
L
R116
Bacterial Count
Layers (µm)
z
z+2
z+4
z+6
z+8
0
50
100
150
200
Day -2
Day 0
Day 2

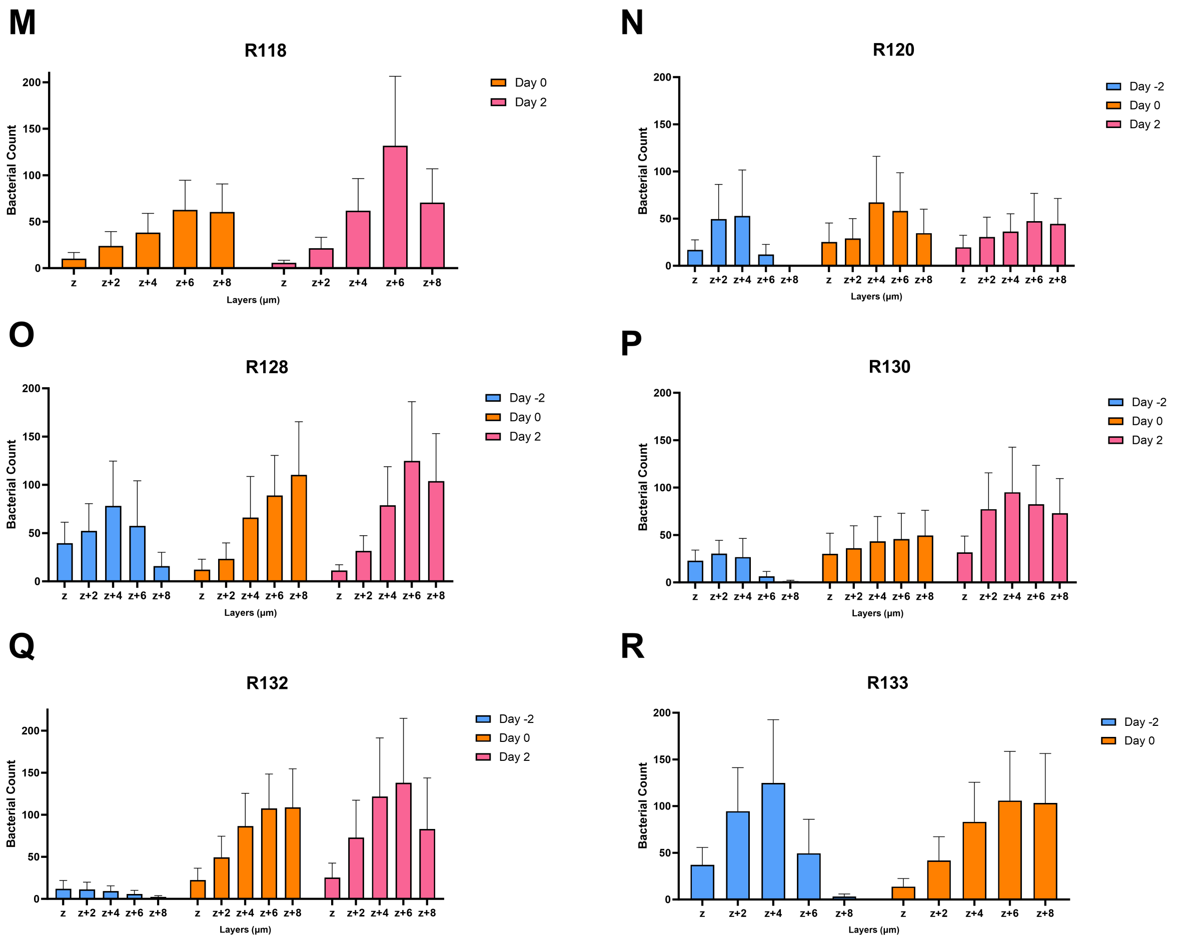

Supplemental Figure 4. Total bacterial counts among the layers of BV biofilm at each time point of interest [2 days pre-iBV (Day -2), day of iBV (Day 0), and 2 days post-iBV (Day +2)] in 18 individual iBV case participants (Figures A-R). Day -2 is labeled in blue, Day 0 in orange, and Day +2 in pink. Some iBV cases do not have data available for each timepoint (e.g., Figures F, J, M, and R). Layer z represents the bottom layer of the BV biofilm. Error bars represent the standard error of the mean.

Supplemental Table 1. Meta-data obtained from participants on study surveys and daily diaries.

Recorded Characteristics for Participants	
**Enrollment Survey**	**Daily Diary**
Age, Race, Ethnicity	Sexual Activities
Education	Currently Menstruating
Douching	Douching
Vaginal Symptoms	Vaginal Symptoms
Tobacco, Alcohol, Drug Use	Tobacco, Alcohol, Drug Use
Contraception Use	Contraception Use
Sexual History/STI History	Antibiotic Use
Date of Last Menstrual Period	

Variables were repeated in the study surveys and daily diaries to track changes over time.
